# Extraction, purification, structural features and biological activities of longan fruit pulp (Longyan) polysaccharides: A review

**DOI:** 10.3389/fnut.2022.914679

**Published:** 2022-07-25

**Authors:** Xuan Yue, Zhejie Chen, Jinming Zhang, Chi Huang, Shiyi Zhao, Xuebo Li, Yan Qu, Chen Zhang

**Affiliations:** ^1^State Key Laboratory of Southwestern Chinese Medicine Resources, School of Pharmacy, Chengdu University of Traditional Chinese Medicine, Chengdu, China; ^2^State Key Laboratory of Quality Research in Chinese Medicine, Institute of Chinese Medical Sciences, University of Macau, Macau, China

**Keywords:** longan fruit pulp polysaccharide, extraction, structures, bioactivities, immune regulation, antitumor

## Abstract

*Dimocarpus longan Lour*. (also called as longan) is a subtropical and tropical evergreen tree belonging to the Sapindaceae family and is widely distributed in China, Southeast Asia and South Asia. The pulp of longan fruit is a time-honored traditional medicinal and edible raw material in China and some Asian countries. With the advancement of food therapy in modern medicine, longan fruit pulp as an edible medicinal material is expected to usher in its rapid development as a functional nutrient. As one of the main constituents of longan fruit pulp, longan fruit pulp polysaccharides (LPs) play an indispensable role in longan fruit pulp-based functional utilization. This review aims to outline the extraction and purification methods, structural characteristics and biological activities (such as immunoregulatory, anti-tumor, prebiotic, anti-oxidant, anti-inflammatory and inhibition of AChE activity) of LPs. Besides, the structure-activity relationship, application prospect and patent application of LPs were analyzed and summarized. Through the systematic summary, this review attempts to provide a theoretical basis for further research of LPs, and promote the industrial development of this class of polysaccharides.

## Introduction

**Figure 1:**
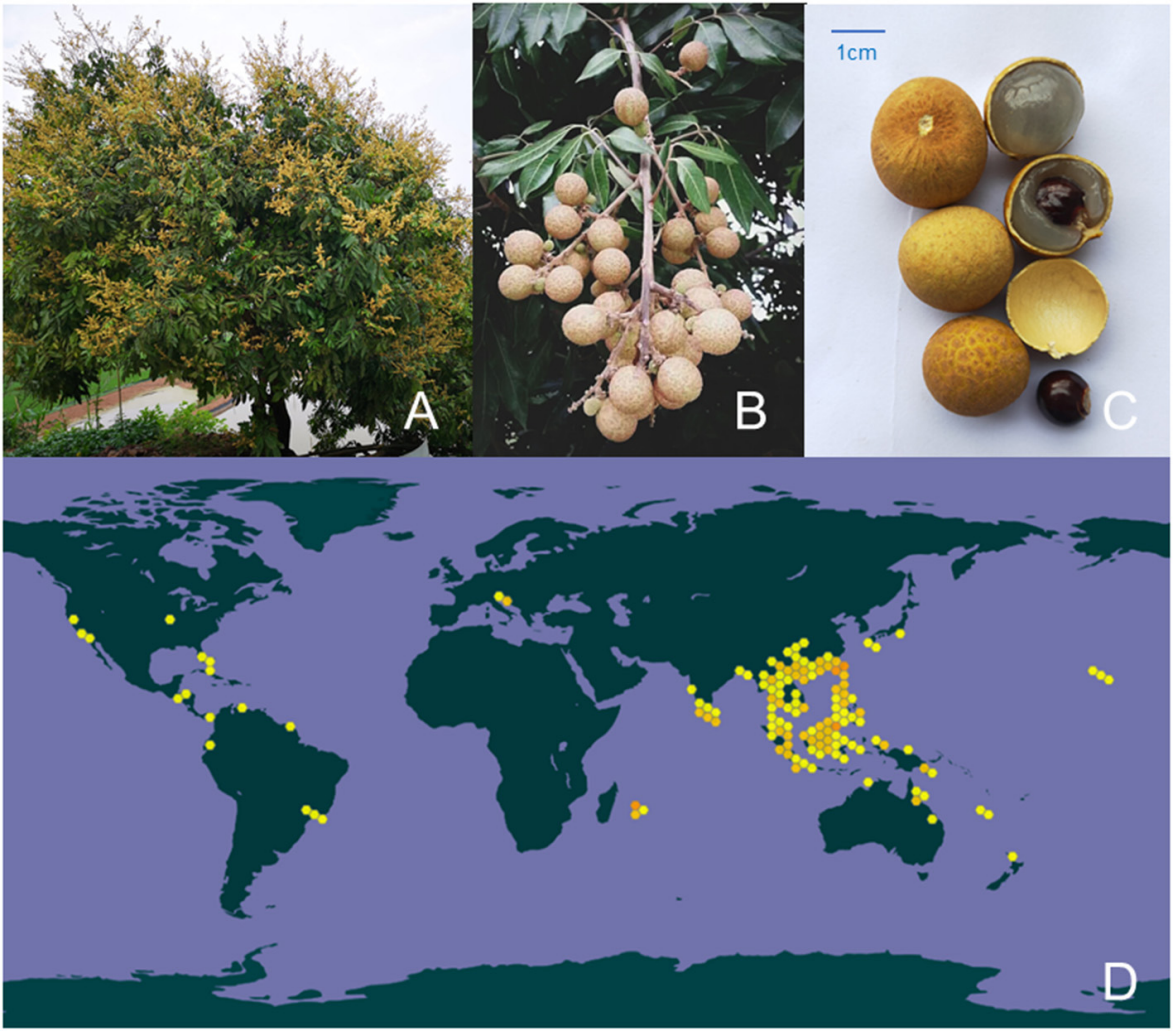
Botanical characteristics and the distribution of longan in the world. **(A)** The plant of longan, **(B)** the fruit of longan, and **(C)** different parts of longan fruit. **(D)** The approximate distribution of longan in the world (Data from the Global Biodiversity Information Facility, color figure can be viewed at wileyonlinelibrary.com).

*Dimocarpus longan Lour*. (also called as longan) is a subtropical and tropical evergreen tree widely distributed in warm climates around the world, and mainly concentrated in southern China, Southeast Asia and South Asia, especially in China where longan has been cultivated for more than 2,000 years. Longan trees are mostly cultivated on embankment and gardens, and their fruits are generally picked in autumn. Longan fruits are perishable, have a short storage period, and are susceptible to post-harvest diseases. Longan fruit is divided into pulp, pericarp and core. Modern research focuses on longan fruit pulp, longan fruit pulp polysaccharides (LPs) are the main components of the water extract of longan fruit pulp (Figure 1). In Chinese traditional medical and botany classics, such as Shennong's Classic of Materia Medica (Simplified Chinese: 神農本草經) and the Nanfang caomu Zhuang (Simplified Chinese: 南方草木狀), the precursory medical scientists and botanists have deeply summarized the medicinal and nutritive values and related cultivation history of longan fruit pulp (fruit flesh). Longan fruit pulp can be eaten fresh and dried, it has been demonstrated that the pulp of longan fruit is rich in dietary fiber, vitamins, phosphor, protein, polysaccharides and minerals, which contributes to the high nutritional value and the beneficial effect of spleen and brain (1). Based on the theory of food and drug homology, Chinese medicine practitioners have concluded through long practice and experience that the nutritional value of longan fruit pulp provides an irreplaceable premise for it to become an outstanding tonic. Generally, the dried longan fruit pulp (also known as Guiyuan) is combined with other herbs to treat diseases caused by body deficiency, such as Guipi Decoction and Jade Ling Ointment. Besides, in other Asian countries such as Thailand, Vietnam and Indonesia, longan fruit pulp is also employed as a food therapy material for amnesia, insomnia, neurasthenia, heart palpitations and fatigue of traditional medicines (2).

As a tonic, longan fruit pulp is rich in nutritional value, among which longan fruit pulp polysaccharides (Usually denoted as “Longan polysaccharides,” abbreviated as LPs), as one of the material bases of longan fruit pulp health effects, have been proved to have immunomodulatory, anti-tumor and anti-oxidant physiological effects. So far, with the gradual improvement of separation and purification technology and the progress of biomedical research, scholars have conducted extensive research on the structural characteristics and biological activities of LPs. LPs are heterogeneous polysaccharides mainly composed of glucose (Glc), galactose (Gal), mannose (Man), rhamnose (Rha), ribose (Rib), glucuronic acid (GlcA) and galacturonic acid (GalA). Crude LPs were extracted from longan fruit pulp by different extraction methods, and then further purification could yield relatively pure and homogeneous LPs. In addition, the modification of polysaccharides has been a hot topic in modern polysaccharide research. In order to improve the biological activity of LPs and broaden their applications, scholars have also modified LPs according to their structures, such as chemical modifications like sulfation and phosphorylation, and biological modifications like enzymatic modifications and fermentation modifications of LPs. In addition, pharmacological studies of LPs have been carried out in an orderly manner, showing a variety of biological activities, such as LPs can enhance the ability of scavenging free radicals, immunomodulation, anti-tumor, and also exert the ability to regulate the dynamic balance of intestinal flora and enhance memory. For example, polysaccharides extracted from longan fruit pulp were shown to result in better learning and memory in mice by passive avoidance tests (3). Therefore, LPs have great commercial potential for functional food development, disease adjuvant therapy and cosmetics development.

**Figure 2:**
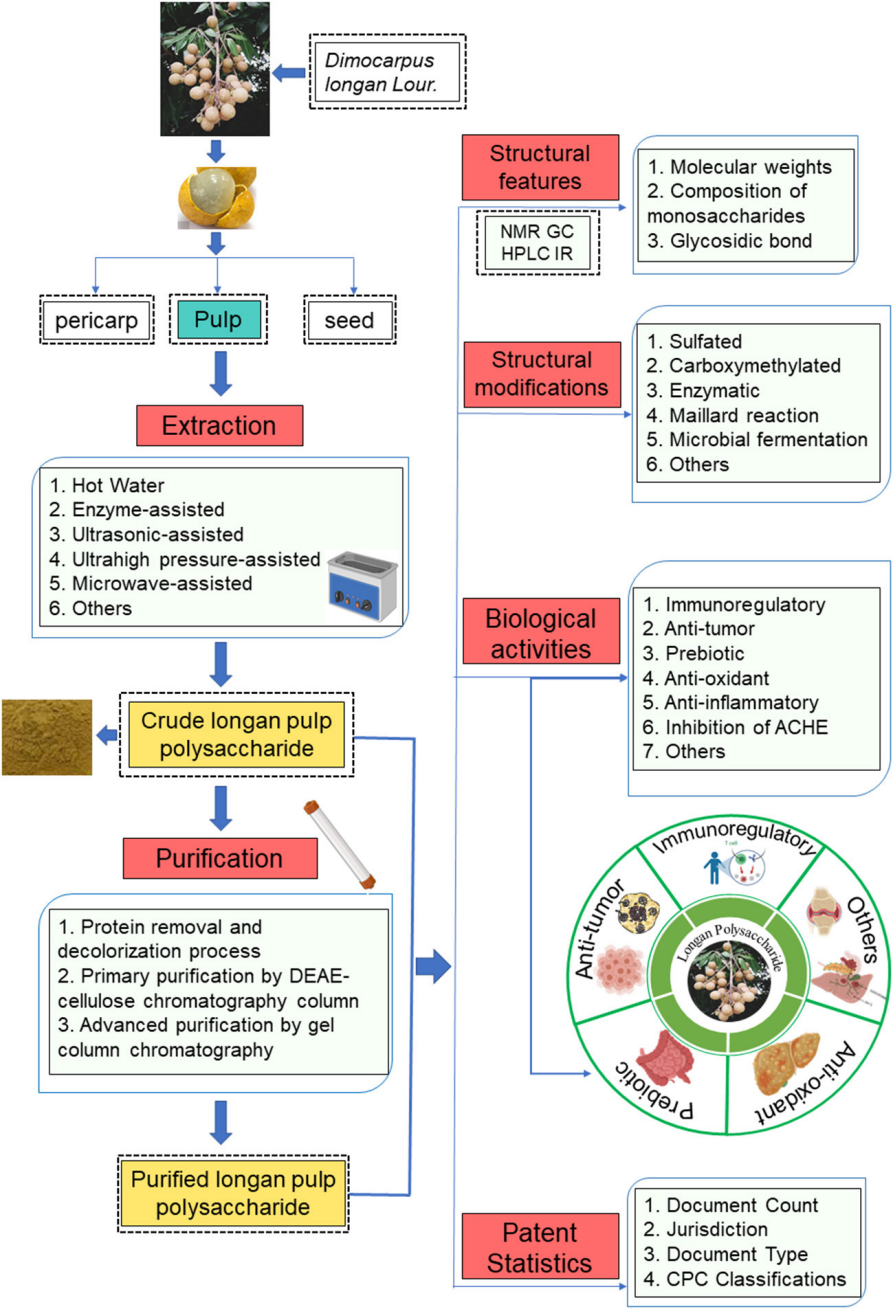
Schematic diagram of extraction, purification, basic structure, structural modification, biological activity and patent statistics of LPs.

To date, there are plenty of studies on LPs, but there is no systematic review on LPs. The purpose of this paper is to comprehensively summarize the extraction and purification methods, structural characteristics, and multiple biological activities (e.g., immunomodulatory, anti-tumor, prebiotic, anti-oxidant, anti-inflammatory, and AChE inhibitory activities) of LPs. Besides, the structural modifications, patent statistics as well as the application prospects of the LPs are also summarized (Figure 2). These contents provide the theoretical basis for the further study of LPs and will contribute to the further diversified industrial development of LPs.

## Methods of extraction and purification

### Extraction

The extraction of LPs is the first step in their basic research and commercial application, and our qualitative and quantitative studies of their active ingredients depend mainly on the choice of extraction methods. In general, the extraction process of LPs has three main steps: (1) Removal of pericarp, core and other impurities from longan fruit; (2) Disruption of the cell wall of longan fruit pulp cells, and the results of studies on plant cell walls indicated that plant polysaccharides are one of the main structural components of plant cell walls, so the extraction method that can effectively decompose the cell wall and release LPs without destroying the polysaccharide structure as much as possible should be selected (4); (3) Determination of the actual extraction rate, content and structure of LPs. At present, the extraction methods of LPs are generally mainly conventional extraction methods such as hot water method, microwave method, ultrasonic method and enzymatic method. In contrast, ultra-high pressure (UHP) technology and auxiliary extraction methods such as microwave-assisted extraction (MAE), ultrasonic-assisted extraction (UAE), ultra-high pressure (UHP)-assisted enzymes and ultrafine crushing-assisted enzymes can improve the original extraction process to a certain extent and obtain higher extraction efficiency. The researchers investigated the extraction temperature, solvent pH, extraction time and material-to-liquid ratio in LPs extraction methods to obtain the conditions for maximum extraction rate.

#### Hot water extraction

HWE is the most widespread and simple extraction method. Under high temperature water environment, plant cell walls are softened and polysaccharides are easily released and solubilized. The HWE method has the advantages of good permeability, high leaching rate and simple operation scheme, but it also has disadvantages such as time consuming, high temperature requirement, easy starch pasting and easy polysaccharide hydrolysis (5). Usually, prior to HWE of LPs, dried longan fruit pulp was crushed with a high-speed grinder and then the sample was pre-treated with a solvent (e.g., ethanol, acetone, or methanol) to remove low molecular weight (Mw) substances such as pigments, monosaccharides, and oligosaccharides. Distilled water (1:20 to 1:40, W/V) was used to extract the longan fruit pulp at 50°C to 90°C for 90–240 min, followed by filtration to obtain the extract, and the filtrate was concentrated to a certain amount under reduced pressure at no more than 65°C. Then, a certain amount of anhydrous ethanol was added to make the polysaccharides precipitate out as much as possible, and the crude LPs were precipitated after being stored at 4°C for 12 h. The content of LPs was determined by phenol-sulfuric acid method. For example, Zhong et al. optimized the HWE process of LPs by response surface methodology (RSM), and they concluded that the yield of LPs could reach 0.413% under the optimal conditions of liquid to solid ratio of 4:5, and extraction time of 4.5 h (6). However, many water-soluble non-polysaccharides were also dissolved in solution during HWE of LPs. So, the crude LPs obtained during this process need to be further purified.

#### Enzyme-assisted extraction

Enzyme technology is a biotechnology that has been widely applied to the extraction of active ingredients in recent years. In the extraction of LPs, the use of enzymes can temper the extraction conditions and accelerate the release of LPs under relatively mild conditions. This is mainly because pectin, cellulose and hemicellulose in longan fruit pulp are the main cell wall polysaccharides that play an important role in maintaining the texture of ripe fruits, however, EAE can break the cell wall by specifically catalyzing the degradation of these cell wall polysaccharides, thus releasing LPs in large quantities and achieving improved extraction efficiency (7). Therefore, the strategy of EAE for effective enrichment of LPs is characterized by high extraction rate, simple operation and high reproducibility (8, 9), and commonly used enzymes include cellulase (10, 11), dextranase, amylase, papain, hemi-cellulase and pectinase (12). It is important to note that when extracting LPs, enzymes are usually added during maceration, when enzymatic reactions (enzymatic dissolution of cell surface structures and intercellular associations) are most likely to occur, followed by HWE in order to achieve polysaccharide solubilization. Cheng et al. produced transformed longan fruit pulp with low sucrose content and rich in Fructo-oligosaccharide (Fos) by treating fresh longan fruit pulp with 300 mg of complex plant hydrolase Viscoozyme L at pH = 6.0 and 55°C. Viscoozyme L has potent arabinose hydrolase and pectinase activities and can be used to degrade such cell wall polysaccharides to release water-soluble LPs. The results of content determination showed that the content of water-soluble LPs obtained from the enzymatically treated extracts increased significantly from 3.61 to 8.30 mg/g, while the insoluble fiber content decreased significantly with increasing enzymatic digestion time. This was because during enzymatic digestion, a large amount of insoluble fiber could be hydrolyzed into oligomers, which are recycled into the water-soluble LPs group (13). However, enzymatic digestion of LPs has some disadvantages, such as some enzymes are expensive and not easily available; strict control of temperature and pH is required during the experimental process, and changes in conditions may lead to inactivation of the enzymes; and may also destroy the advanced structure of some LPs.

#### Ultrasonic-assisted extraction

In recent years, UAE has been widely used for the extraction of LPs. The enhanced extraction efficiency of ultrasound is mainly attributed to its mechanical and cavitation effects, the former greatly facilitating mass transfer between immiscible phases through hyper-stirring, especially at low frequencies. The latter can disrupt the cell wall by cavitation, resulting in the reduction of particle size and enhanced mass transfer through the cell wall (14). Compared with the conventional HWE method, UAE can better improve the extraction efficiency, save operation time, simplify the operation process, and depolymerize polysaccharides to a limited Mw. Based on the combination of artificial neural network and genetic algorithm, Yang et al. derived an optimal condition for ultrasonic extraction of LPs at 120 watts, 12 min and 57°C (15). In addition, Zhong et al. employed ultrasonic technique to extract LPs from dried longan fruit pulp. On the basis of single factor experiment, the optimal extraction conditions for LPs were ultrasonic power of 680 W, extraction time of 4.5 min, and water-to-material ratio of 25 mL/g. At this time, the yield of LPs was 4.455 ± 0.093%, which is in good agreement with the predicted value of 4.469% (16). Notably, UAE led to some changes in the structure and properties of LPs to some extent, depended on the parameters of applied power and operating conditions, so further studies on the UAE of LPs are needed (17).

#### Ultrahigh pressure (UHP)-assisted extraction

UHP-assisted extraction is an emerging extraction technique developed on the basis of food UHP technology, and modern research has applied it to the extraction of polysaccharides. The UHP-assisted extraction efficiency of LPs was greatly improved due to the rapid pressure changes in a short period of time that accelerated the rupture of cell walls and enhanced the release of intracellular products and the penetration of solvents into cells. Thus, UHP-assisted extraction achieved high yield of the target product with reduced processing time, temperature and energy input, which is efficient, time-saving and environmentally friendly (18). Bai et al. to consider the yield and structure of LPs as well as to facilitate practical extraction, they optimized the conditions of UHP-assisted enzymatic extraction of LPs by RSM and analytical design. The optimized conditions were: water to pretreatment material ratio of 42 mL/g, enzyme to pretreatment material ratio of 1:100, enzymatic digestion time of 1.7 h, and ultra-high pressure of 407 MPa for 6 min, which resulted in a yield of 8.55% of LPs (3). However, scholars have noticed that the yield of water-soluble LPs decreases instead after a period of UHP treatment, which may be due to the excessive depolymerization of the structure of LPs as the holding time increases, and their yield decreases as a result. But it was undeniable that the extraction rate of total polysaccharides from longan fruit pulp was still largely enhanced after UHP-assisted extraction within the suitable conditions (18).

#### Microwave-assisted extraction

MAE is a combination of microwave and traditional solvent extraction methods that directly converts microwave energy into thermal energy through simultaneous molecularly occurring dual polarization and ionic conduction, allowing the sample to be heated almost immediately, thus extracting bioactive compounds from the material matrix into solution (19). Thus, MAE of polysaccharides has the significant advantage of simultaneously increasing their yield and bioactivity (20). Li et al. used the microwave pretreatment-HWE method to extract LPs. Within the investigated experimental range, they arrived at the optimal extraction process conditions for LPs by single-factor investigation and orthogonal experiments as follows: the microwave pretreatment power was kept at 700 W, the treatment time maintained at 60 s, HWE material-liquid ratio controlled at 1:15, extraction temperature at 100°C, extraction time at 7 h, and stirring speed at 240 r/min, when the yield of LPs can reach 9 mg/g (21). Nevertheless, excessive microwave power and other factors could cause rapid heating of longan fruit pulp cells and then severed water loss, resulting in the destruction of the structure and activity of LPs.

#### Others

Superfine grinding is an emerging technology that refers to the preparation of ultrafine powders with small solid particles (1 nm−100 mm) and good surface properties, which has the advantages of time saving, solvent saving and energy saving. After the pulp of longan fruit was ultra-micronized, the size of the extracted LPs particles decreased and the surface area increased, and their adsorption and water permeability were enhanced, which eventually led to the better dissolution of LPs in the solvent and the extraction efficiency was effectively enhanced (22). It is worth noting that modern research on the extraction process of LPs focuses on the dynamic balance of yield and activity. All current extraction methods effectively improve the extraction rate of LPs, but the treatments taken during the extraction process (e.g., high temperature, pH, high pressure, ultrasonic and microwave) may lead to changes in the structure of LPs, which in turn may affect their biological activity. Therefore, the research on the extraction methods of LPs needs to be further strengthened.

### Purification

As with other plant polysaccharides, crude LPs obtained through the extraction process usually also contain impurities such as proteins, nucleic acids, small molecules and other compounds. Therefore, further purification is required to remove these compounds. According to the literature, the main methods for the deproteinization of plant polysaccharides are the trifluoro-trichloroethane method, the trichloroacetic acid method, the Sevag method, the bio-enzymatic method, and a combination of several methods. Among them, the application of Sevage reagent is considered as one of the most common strategies to remove crude protein from LPs because it can be used repeatedly to remove additional protein residues. However, the Sevag deproteinization method also has disadvantages such as time-consuming, severe loss of LPs, and the organic reagents used tend to cause structural changes of LPs. Moreover, the acid precipitation method can cause degradation of some polysaccharides, so that the yield of LPs is affected. Although the protease method is safe, the enzymatic digestion is usually insufficient and the degree of refinement is low, therefore, researchers usually use a mixture of several methods for better protein removal. Modern studies have also shown that small pigment molecules are often adsorbed by activated carbon; Macroporous resins can effectively remove pigments and proteins from LPs with mild conditions and simple processes, and also help to maintain the activity of LPs; Column chromatography is the most widely applied in the purification of LPs because of its simple operation, good purification performance and a wide variety of purifiable substances; In addition, there are also a few reports on the separation and purification of bioactive polysaccharides by high-speed counter-current chromatography (23).

Usually, LPs obtained by deproteinization and decolorization processes also contain polysaccharide fractions of various properties. Therefore, in order to obtain single polysaccharide fractions with different molecular weights, we need further purification. LPs are macromolecular carbohydrates consisting of aldose or ketose, which are weakly acidic and can be adsorbed by ion exchange with anion exchange resins in neutral solutions, but the application of strong alkaline anion exchange resins is limited because LPs are prone to isomerization or decomposition in a strongly alkaline environment. Thus, the diethyl-aminoethyl (DEAE) cellulose column was invented by taking advantage of the property that the cis-dihydroxy group in polysaccharides can form complex salts with boric acid, and was widely applied in the separation and purification process of LPs. However, this method was mainly used for the primary purification of LPs. Gel chromatography is currently the most commonly applied advanced purification method for LPs, and the commonly used gels are dextran gel G100 (Sephadex G100), agarose gel 4B (Sepharose 4B) and propylated dextran gel S-400 (Sephacryl S-400). In the separation and purification process, the gel column acts as a molecular sieve, allowing LPs of different molecular weights to be separated. For instance, Meng et al. used HWE, alcohol precipitation and deproteinization to extract crude LPs. Then separated by DEAE-cellulose anion exchange and Sephacryl S-300 high-resolution (HR) gel filtration chromatography to obtain a single water-soluble polysaccharide LP1 (24). This suggests that the use of a combination of purification methods is indispensable to obtain LPs with relatively well-defined structures.

## Structural features

### Basic structures

**Figure 3:**
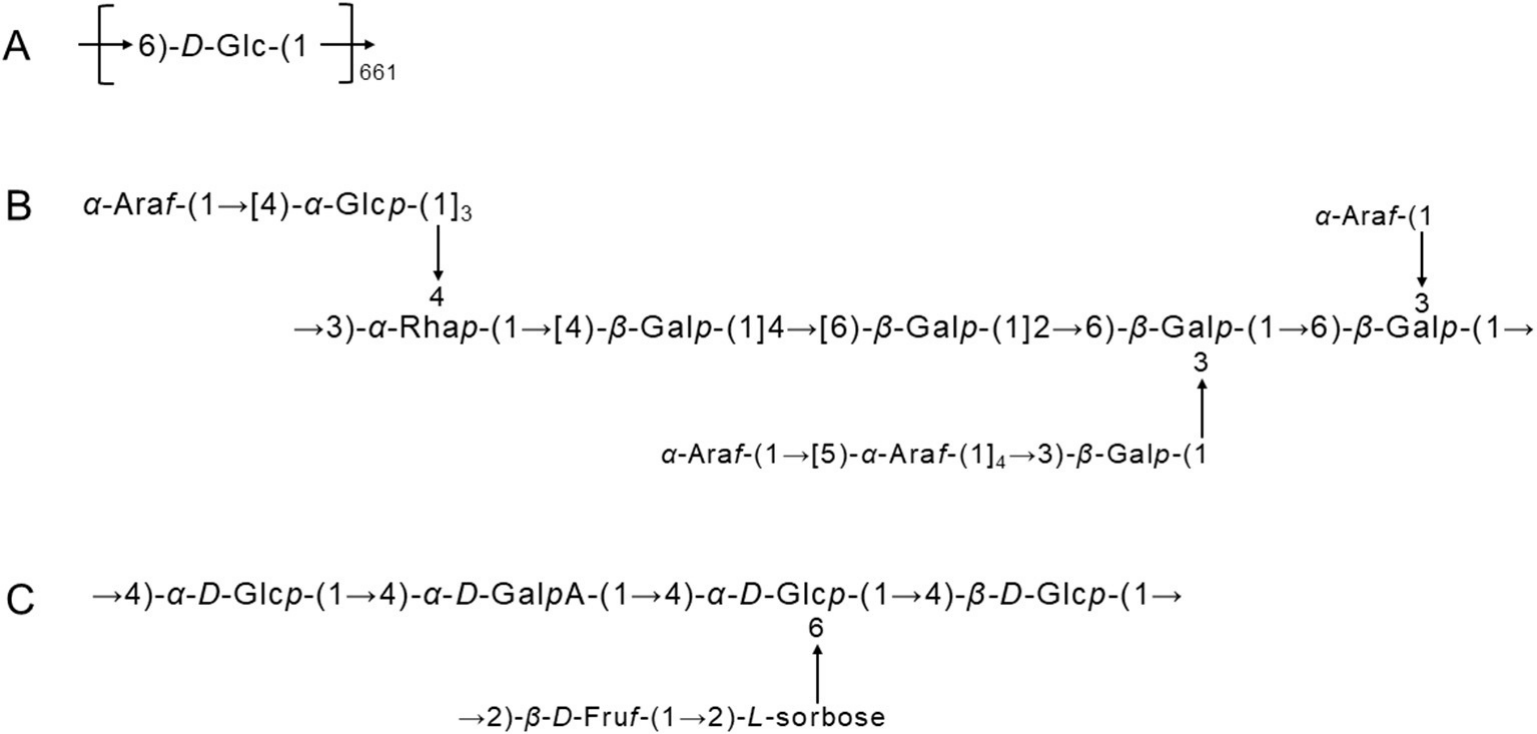
The structures of longan pulp polysaccharides. **(A)** LPS1, **(B)** LPIIa, and **(C)** LP1.

The structural characteristics of LPs are mainly determined by the Mw, the composition and sequence of monosaccharides, the conformation and position of glycosidic bonds, the type of branched chains and the degree of polymerization. Different polysaccharide compositions and structures influence its biological activity (25, 26). So far, dozens of polysaccharide components have been extracted from longan fruit pulp. After the purification of crude LPs, the basic structural characteristics of LPs are generally studied accordingly by partial acid hydrolysis, periodate oxidation, Smith degradation, gel permeation chromatography, high performance liquid chromatography (HPLC), gas chromatography (GC), Fourier transform infrared spectroscopy (FT-IR), gas chromatography-mass spectrometry (GC-MS) and nuclear magnetic resonance (NMR) spectroscopy. Due to the differences in raw materials, extraction and purification processes and other conditions, the results of various reported studies on the structural characteristics and monosaccharide composition of LPs are diverse. Therefore, to clarify the current research status and better grasp the structural characteristics of LPs, we summarized the structure and some chemical parameters of LPs by classification in Table 1, and mapped the structure of homogeneous LPs with more definite Mw extracted in recent years in Figure 3.

**Table 1:** The extraction, purification, and structural characterization of polysaccharides from the longan fruit pulp.

**No**.	**Component name**	**Extraction method**	**Purification method**	**Monosaccharide composition**	**Mw (Da)**	**Structures features**	**Structure-activity relationship**	**References**
1	LPS1	Hot water	Gel filtration and anion-exchange chromatography	Glc (Consists of 661 glucose residues)	1.08 ×10^5^	(1 → 6)-α-D-glucan	Immunomodulatory and anti-cancer	(27)
2	LP-H	Hot water	Sevage	Rha:Ara:Man:Glc:Gal = 1.2:4.6:1:3:2.2	2.38 ×10^5^	Mainly consisted of → 3)-*α-L*-Ara*f*-(1 → , → 3,6)-*β-D*-Gal*p*-(1 →, *α-L*-Rha*p*-(1 →, → 4)-*β-D*-Glc*p*-(1 →	Prebiotic	(22)
3	LP-S	Superfine grinding		Rha:Ara:Man:Glc:Gal = 1.2:4.9:1:2.1:4	2.28 ×10^5^	Mainly consisted of → 3)-*α-L*-Ara*f*-(1 →, → 3,6)*-β-D*-Gal*p*-(1 →, *α-L*-Rha*p*-(1 →, → 4)-*β-D*-Gal*p*-(1 →	Prebiotic	
4	LP-SE	Superfine grinding-assisted enzymatic		Rha:Ara:Man:Glc:Gal = 1.1:4.3:1:1.5:2.8	1.90 ×10^5^	Mainly consisted of → 3)-*α-L*-Ara*f*-(1 →, → 3,6)-*β-D*-Gal*p*-(1 →, *α-L*-Rha*p*-(1 →, → 5)-*α-L*-Ara*f*-(1 →	With better prebiotic	
5	LP-UE	Ultra-high pressure-assisted enzyme	Sevage	Rha:Ara:Xyl:Man:Glc:Gal = 5.8:40.8:1:2.3:32.5:26.7	2.91 ×10^5^	β-type glycosidic bond	Moderate acetylcholinesterase inhibitory	(3)
6	LP-H	Hot water		Rha:Ara:Xyl:Man:Glc:Gal = 1.1:11.9:1:1.6:12.2:11.1	1.19 ×10^5^		Slight acetylcholinesterase inhibitory	
7	LP-U	Ultra-high pressure		Rha:Ara:Xyl:Man:Glc:Gal = 1.2:11.7:1.2:1:10:12.4	1.32 ×10^5^			
8	LP-E	Enzymatic method		Rha:Ara:Xyl:Man:Glc:Gal = 5.9:46.2:1:6:22.2:33.7	3.18 ×10^5^			
9	LP	Unfermented, hot water	Sevage	Rha:Ara:Xyl:Man:Glc:Gal = 5.7:28.5:1:1.5:15.3:13.3	2.22 ×10^5^	Mainly consisted of α-type and β-type glycosidic bond	Immunomodulatory and prebiotic	(28)
10	LP-F	After fermentation, hot water	Sevage	Rha:Ara:Xyl:Man:Glc:Gal = 5.1:26.5:1:1.8:7.8:12.4	1.11 ×10^5^		Stronger immunomodulatory and prebiotic	
11	LPs	Water extraction, alcohol precipitation	Gel filtration chromatography	Man:Rib:Rha:GlcA:GalA:Glu:Gal = 4:1.25:3.75:3:1:25:5.25	(2.13–7.07) ×10^3^	N/A	Immunostimulatory and free radical scavenging	(29)
12	MLPs	Water extraction, alcohol precipitation	Gel filtration chromatography	Man:Rib:Rha:GlcA:GalA:Glu:Gal = 19:1:17:13:3:100:24	2.78 ×10^4^-1.00 ×10^6^	Possess more branched structures	Stronger free radical scavenging abilities and immune-stimulating effects, but weaker growth-inhibitory activities against cancer cells	
13	LP1	Hot water	Sevage, chromatography of DEAE-cellulose and Sephadex G-100	N/A	1.23 ×10^3^	N/A	Immunomodulatory and anti-tumor	(30)
14	LP1-S	Hot water	Sevage, chromatography of DEAE-cellulose and Sephadex G-100	N/A	1.05 ×10^5^	N/A	Immunomodulatory and stronger anti-tumor	
15	LPIIa	Ultrahigh pressure-assisted enzymatic	HiPrep 26/60 Sephacryl S-300 HR column	Rib:Ara:Xyl:Glc:Gal = 1.05:1:22.88:1.01:2.59:34.58	1.59 ×10^5^	The backbone consisted of (1 → 3,4)-α-Rha*p*, (1 → 4)-β-Gal*p*, (1 → 6)-β-Gal*p*, (1 → 3,6)-β-Gal*p*, with branches at the O-4 of Rha and O-3 of Gal, consisting of side chains of α-Ara*f, β*-Gal*p*, and α-Glc*p*	Anti-inflammatory and protective intestinal barrier function	(31)
16	LP3	After extraction with distilled water, cellulase enzymolysis and ultrasonic cell disintegration were used.	Anion exchange resin D301-F	Rib:Rha:Ara:Xyl:Man:Glc:Gal = 4.85:1.06:14.55:1.00:28.36:70.89:8.58	N/A	N/A	Strong immunoregulatory	(32)
17	LPP	N/A	Sephadex G-100 gel column and gel filtration chromatogram	N/A	3.75 ×10^4^	N/A	The combination of FITC pre-labeling and HPSEC-FD makes the quantitative determination of LPP possible in mouse plasma, spleen and lung samples	(33)
18	LPPF	N/A	Sephadex G-100 gel column and gel filtration chromatography	N/A	3.9 ×10^4^	N/A		
19	LPI	N/A	DEAE-cellulose anion-exchange chromatography	Rib:Rha:Ara:Xyl:Man:Glc:Gal = 0.57:0.01:1.00:0.20:9.64:21.84:0.73	1.459 ×10^4^	Mainly consisted of (1 → 6)-*α-D*-Glc*p*, (1 → 5)-*α-L*-Ara*f* and (1 → 4)-*β-D*-Man*p*	Except for LPI, the other three significantly stimulated lymphocyte proliferation in the dose range of 100–400 μg/mL and their stimulations on normal/LPS-induced proliferation and depressions on ConA-Induced proliferation could be ordered as LPIII > LPIV > LPII > LPI. All the fractions had the optimal dose of 100 μg/mL on enhancing macrophage phagocytosis. Among them, LPII had the considerable yield and activity for exploiting as a potential immunoadjuvant	(34)
20	LPII			Rib:Rha:Ara:Xyl:Man:Glc:Gal = 1.00:0.22:3.00:0.21:5.85:14.62:1.77	6.834 ×10^4^	Mainly consisted of (1 → 6)-*α-D*-Glc*p*, (1 → 5)-*α-D*-Ara*f* and *β-D*-Gal*p*		
21	LPIII			Rib:Rha:Ara:Xyl:Man:Glc:Gal = 1.00:3.21:4.70:0.56:0.41:0.66:2.18	1.074 ×10^5^	Mainly consisted of (1 → 4)-*β-D*-Rha*p* and (1 → 5)-*α-L*-Dra*f*		
22	LPIV			Rib:Rha:Ara:Xyl:Man:Glc:Gal = 7.52:7.58:7.69:7.82:9.59:9.70:9.91	5.282 ×10^6^	N/A		
23	LPD2	Hot water	Weak anion exchanger	Ara:Ma:Glc:Gal = 0.25:0.49:1:0.5	9.64 ×10^6^	The main linkages of the sugar residues were (1 → 4)-β-Glc and (1 → 6)-β-Man	Significantly enhanced the lymphocytes proliferation, phagocytosis and NO and IL-6 secretion by macrophage	(35)
24	LPIa	Hot water	Sevage, DEAE-Sepharose Fast Flow chromatography and HiPrep 26/60 Sephacryl S-300 HR chromatography	Rha:Rib:Fuc:Ara:Xyl:Man:Glc:Gal = 0.99:1.37:34.61:1.48:1.73:5.86:55.16	1.47 ×10^5^	Mainly consisted of → 3)-α-Ara*f*-(1 →, → 3,6)-β-Gal*p*-(1 →, α-Ara*f*-(1 → and → 5)-α-Ara*f*-(1 →	Both LPIa and LPIIa have higher intestinal barrier protection and immunoregulatory activities than LPIIIa and LPIVa	(36)
25	LPIIa			Rha:Rib:Ara:Xyl:Glc:Gal = 1.05:1.00:22.88:1.01:2.59:34.58	1.593 ×10^5^	Mainly consisted of → 3)-α-Ara*f*-(1 →, → 3,6)-β-Gal*p*-(1 →, α-Ara*f*-(1 → and → 5)-α-Ara*f*-(1 →		
26	LPIIIa			Rha:Rib:Fuc:Ara:Man:Glc:Gal = 14.46:1.85:2.31:46.17:1.00:1.97:20.99	1.94 ×10^4^	Mainly consisted of → 3)-α-Ara*f*-(1 →, → 3,6)-β-Gal*p*-(1 →, → 3)-β-Gal*p*A-(1 → and α-Rha*p*-(1 →		
27	LPIVa			Rha:Rib:Ara:Man:Glc:Gal = 4.71:0.38:25.03:1.00:2.53:15.50	4.4 ×10^4^	Mainly consisted of → 3)-α-Ara*f*-(1 →, → 3,6)-β-Gal*p*-(1 →, → 3)-β-Gal*p*A-(1 → and α-Rha*p*-(1 →		
28	LPPMs	Alkali-extraction and acid-precipitation	N/A	N/A	N/A	N/A	Anti-oxidation, anti-tumor and immune stimulation activity were enhanced	(37)
29	LP1			Rib:Rha:Ara:Xyl:Man:Glc:Gal = 6.1:4.9:52.2:1:11.1:72.2:20.3		N/A	The immunoregulatory activity was weaker than that of LP2 and LP3	(38)
30	LP2	Ultrasound-assisted enzymatic method	Gel column chromatograms	Rib:Rha:Ara:Xyl:Man:Glc:Gal = 2:1:16.7:23.7:57.2:114.3:2.2	>10^5^	Mainly composed of glucosyl residues in the α-pyranose form	Immunoregulatory activity	
31	LP3			Rib:Rha:Ara:Xyl:Man:Glc:Gal = 11.7:1:33.3:4.7:213.3:472:13.3			Immunoregulatory activity	
32	LP1	Hot water extraction and alcohol precipitation	DEAE-cellulose anion-exchange and Sephacryl S-300 HR gel chromatography	Glc:GalA:Ara:Gal = 5.39:1.04:0.74:0.21	1.16 ×10^2^	Consisted of a backbone of → 4)-*α-D*-Glc*p*-(1 → 4)-*α-D*-Gal*p*A-(1 → 4)-*α-D*-Glc*p*-(1 → 4)-*β-D*-Glc*p*-(1 → units with poly saccharide side chains composed of → 2)-*β-D*-Fru*f*-(1 → 2)-*L*-sorbose-(1 → attached to the O-6 position of the *α-D*-Glc*p* residues	Natural anti-tumor agent with immunomodulatory activity	(24)
33	LPIIa	Hot water	Ion exchange chromatography, gel filtration chromatography	Glc:Ara:Man:Gal = 7.55:1.45:1.22:1.00	4.47 ×10^4^	Mainly composed of → 6)-Glc-(1 →, → 5)-Ara-(1 →, → 4)-Man-(1 → and → 6)-Gal-(1 →	Strong immunoregulatory	(39)
34	LPS-N	Hot water-assisted microwave pretreatment and ethanol precipitation method	DEAE- Cellulose anion exchange chromatography	Xyl:Glc = 1:1.9	1.38 ×10^4^	Belong to β-type heteropolysaccharide with pyran group	N/A	(40)
35	LPS-A1			Rha:Xyl:Ara:Gal = 1:1.64:4.33:2.28	1.382 ×10^3^			
36	LPS-A2			Only Rha	5.71 ×10^5^			
37	LWP	Longan juice ferments	Ultrafiltration	Glc:Man:Gal:Ara:GalA: GlcA = 167.72:3.38:3.13:3.46:2.33:1	(1–3) ×10^4^	Mainly composed of β-type	Hypoglycemic activity and free radical scavenging	(41)
38	LPsx	Hot water extraction and alcohol Precipitation	HPGPC system coupled with Ultrahydragel columns 500 and 250 gel columns at 60°C	Glu:Ara:Gal:Man:Xyl = 95.9:2.1:1.0:0.6:0.4	4.102 ×10^3^	Mainly composed of (1 → 6)-*α-D*-Glu and (1 → 6)-*β-D*-Glu, branched with *α-D*-Glu-(1 →	The immunomodulatory activity study showed that LPsx significantly increased the phagocytosis of macrophages, and strongly promoted the production of NO, IL-1β, IL-6 and TNF-α. Moreover, LPsx could inhibit the inflammatory response induced by lipopolysaccharide.	(42)

Polysaccharides are monosaccharide polymers linked by glycosidic bonds and contain one or more different monosaccharide units. Most of the LPs reported so far are heteropolysaccharides, which have molecular weights ranging from 8.7 to 4800 kDa and contain roughly 9 different ratios of monosaccharides, namely glucose (Glc), galactose (Gal), arabinose (Ara), rhamnose (Rha), mannose (Man), xylose (Xyl), fucose (Fuc), fructose (Fru) and ribose (Rib), and possibly glucuronic acid (GlcA) and galacturonic acid (GalA) as well (43). Their specific structural information is mentioned in some literature. For example, Zhu et al. extracted a water-soluble LP from longan fruit pulp and obtained a pure polysaccharide with a calculated molecular weight of 108 kDa and 661 glc residues by column purification, and named LPS1 (Figure 3A). Chromatographic analysis showed that LPS1 is a high polysaccharide of glc with a glycosidic bond of → 6)-*D*-Glc-(1 → ; NMR spectroscopy pointed that the configuration of the anomeric carbon in the glc residue is α-type; C6 chemical shift further confirmed that the structure of LPS1 is (1 → 6)-α-*D*-glucan (27). Bai et al. purified four acidic polysaccharides from LPs, named LPIa, LPIIa, LPIIIa and LPIVa, whose average molecular weights and polysaccharide compositions can be seen in Table 1. The structural resolution of LPIa indicated that LPIa has a higher Mw and, in particular, contains two other specific glycosidic bonds → 5)-α-Ara*f*-(1 → and α-Ara*f*-(1 →, as well as a complex reticulated surface structure. These may be the main reason for the better biological activity of LPIa than the other three LPs (36). Notably, they also elaborated the detailed structure of LPIIa, in which there are branches at Gal-O-3 and Rha-O-4, and the branches consist of side chains of α-Glc*p*, α-Ara*f* and β-Gal*p* (Figure 3B) (31). In addition, a new water-soluble polysaccharide (LP1) was successfully purified from longan fruit pulp by DEAE-cellulose anion exchange and S-300 HR gel chromatography. The chemical structure was determined by IR, GC and NMR analysis (Table 1) and its detailed structure was mapped (Figure 3C) (24). After purification of LPs by DEAE-cellulose anion-exchange chromatography, Yi et al. obtained 4 fractions of crude LPs, including 1 neutral polysaccharide (LPI) and 3 acidic polysaccharides (LPII-IV), each with different monosaccharide composition, Mw and structural characteristics. The solution properties of the 4 fractions were analyzed by size exclusion chromatography combined with laser scattering (SEC-LLS), viscometry measurement, Congo red complex formation and atomic force microscopy. The study showed that LPI has both a spherical conformation and a triple helix structure, while LPII-IV exists in the form of flexible chains (34). The corresponding structural analysis is shown in Table 1.

Meanwhile, it has been demonstrated that the different structures of LPs largely depend on the different methods of extraction and purification (44). For example, Huang et al. extracted LPs by hot water, superfine grinding and superfine grinding-assisted enzymatic treatment, and obtained LP-H, LP-S and LP-SE, respectively. Compared with LP-H and LP-S, although the Mw, apparent viscosity, particle size and glucose content of LP-SE decreased to a certain extent, the yield, solubility, sugar content, arabinose and mannose content of LPs were increased. All three LPs contained similar glycosidic bonds of α-*L*-Rha*p*-(1 →, → 3)-α-*L*-Ara*f*-(1 → and → 3,6)-β*-D*-Gal*p*-(1 →, while each of them had a specific glycosidic bond of → 4)-β*-D*-Glc*p*-(1 →, → 4)-β*-D*-Gal*p*-(1 → and → 5)-α*-L*-Ara*f*-(1 →, respectively (22). Besides, there are many other relevant examples. We summarized all the structures of LPs reported in Table 1. These studies will provide a theoretical basis for the functional development and potential utilization of LPs.

### Structural modifications

LPs as natural plant polysaccharides have many excellent bioactivities, and the expression of these bioactivities is closely related to their structures. To further improve the physicochemical properties and bioactivities of LPs and enhance the application properties of LPs, researchers have carried out structural modifications of LPs. Commonly, various physical, chemical and biological means are used to modify the structure of LPs (45). It has been suggested that the biological activities of LPs, such as immunomodulatory, anti-viral, anti-inflammatory and anti-tumor, were enhanced after structural modification (46). Therefore, structural modifications based on activity optimization are particularly significant for the study of LPs (29). The existing structural modification methods of LPs were classified and summarized as follows, specific details can be found in Table 2.

**Table 2:** The modification approaches, containing reaction conditions, reaction principles and typical structures of modification products for different structural modifications.

**No**.	**Structural modification method**	**Specific reaction and reaction principle**	**Reaction conditions/specific reaction methods**	**Determination of the degree of modification**	**Typical structures of the modification products (specific features of the structures and biological activities can be found in Table 1)**	**References**
1	Sulfated modification	Concentrated sulfuric acid method - using the principle of sulfuric acid esterification reaction, concentrated sulfuric acid de-hydroxylation, alcohol de-hydrogenation in polysaccharides for the reaction, the introduction of sulfuric acid groups in polysaccharides, its reversible reaction.	Briefly, the mixture of concentrated sulfuric acid and butanol complex (3:1) was prepared in ice bath. Then, 125 mg ammonium sulfate was added and stirred for 10 min at temperature 0 °C (ice-water bath). LP1 (500 mg) was then dripped in slowly. The reaction mixture was stirred for 3 h at 10°C and was then neutralized with sodium hydroxide solution and precipitated with 95% ethanol. The sediment was redissolved with water, followed by dialysis, and freeze-dried.	Barium sulfate turbidimetry method	LP1-S LYRP4	(30, 47)
2	Carboxymethylated modification	Sodium hydroxide-monochloroacetic acid (MCA) method - Introduction of carboxymethyl group on the -OH of polysaccharide by this substitution reaction system.	Dissolve LPs in alkaline solution, add a certain concentration of monochloroacetic acid (MCA), react for a period of time at a specific temperature, then dialyze the reaction solution after adjusting it to neutral, and the solution is concentrated, alcoholic precipitation, and vacuum dried.	UV spectrophotometry	CM-LYP CM-LYP2	(48, 49)
3	Enzymatic modification	Enzymatic reaction -Certain structures can be recognized for catalytic degradation/cleavage.	Firstly, the appropriate enzyme was selected and its optimal reaction temperature and pH were determined; then a certain amount of longan fruit pulp was reacted in the vessel for a period of time and the treated sample was heated in a 90°C water bath for 3 min immediately after the reaction to stop the enzyme reaction. Finally, extraction or collection was performed.	The content of water-soluble polysaccharides was determined using the phenol-sulfuric acid colorimetry (Glucose was used as the standard). The content of soluble pectin was determined by the spectrophotometry method.	Fructo-oligosaccharides (Fos) such as 1-kestose and nystose	(50)
				Quantitative analysis of ethanol-soluble sugars using HPLC. Determination of reducing sugars by UV spectrophotometry.		
4	Maillard reaction modification	Carbonyl ammonia reaction - based on the reduction between the carbonyl group of the sugar and the amino group of the amino compound.	LPs and amino acids were dissolved together in 100 mL NaOH solution (pH 9.0) to the final concentration of 2.5 mg/mL. Ten milliliters of the solution were added into a 25 mL penicillin bottle and sealed. The bottles were placed in a 100°C water bath for 1–6 h, and then transferred to an ice-water bath to end the MR between LPs and amino acids.	The browning degree was determined by the 420 nm absorbance; the Mw distribution was analyzed by a high-performance size exclusion chromatography (HPSEC) method.	MLPs (LPs-Lys)	(29, 37)
5	Microbial fermentation modification	Enzymatic reactions - Lactic acid bacteria or other microorganisms can affect polysaccharide composition by secreting different carbohydrate enzymes. These enzymes can degrade the insoluble pectocellulosic cell wall of fruit into soluble polysaccharides, resulting in increased yield of water-soluble polysaccharides as well as changes to Mw and monosaccharide composition of the extracted polysaccharides.	Fermentation experiments were conducted in aluminum foil sealed Erlenmeyer flasks, each containing 100 mL of pasteurized longan juice without any supplementary nutrients. Inoculum (1 mL) containing 7.0 log cfu/mL of activated *Lactobacillus fermentum* was added to each of the Erlenmeyer flasks. Fermentation was then allowed to proceed at 37 °C for 72 h. Samples were taken at 0, 6, 12, 24, 48 and 72 h for viable cell count, pH, and polysaccharides extraction.	Neutral polysaccharide content was determined using the phenol-sulfuric acid method (Glucose was used as the standard). Protein concentration was determined using the Bradford assay with a bovine serum albumin standard curve.	LP-F (*Lactobacillus fermentum*)	(28, 41)
				Uronic acid content was determined using a modified m-hydroxydiphenyl method with galacturonic acid standards. The monosaccharide composition was determined using (GC-MS).		
6 Others	Alkali dissociation	Depolymerization reaction - sodium hydroxide could depolymerize polysaccharides with compact and high-organized conformation, to obtain its derivatives. Their activity could be enhanced by increasing chain stiffness	50 mg LPI was dissolved in 50 mL distilled water, then 50 mL of NaOH solution was added and the pH was neutralized with 5 mol/L hydrochloric acid solution after alkaline dissociation at room temperature for 10 min. The mixture was dialyzed, concentrated and lyophilized.	The molecular conformations were examined by size exclusion chromatography combined with multi-angle laser light scattering (SEC-MALLS), Congo red test and atomic force microscopy (AFM).	LPI1 and LPI2	(51)
	Phosphorylation modification	Esterification reaction - Phosphorylation modification of LPs using POCl3 (phosphorus oxychloride) as an esterifying agent	The dissolved LPs solution was slowly added to the POCl_3_-pyridine mixture for 60 min (kept in a reaction bath at 0°C or in an ice-water mixture environment), and at the end of the reaction, the appropriate amount of sodium hydroxide solution was added to adjust to neutral. Finally, dialysis, alcohol precipitation and drying under reduced pressure were performed.	Phosphomolybdate blue spectrophotometric determination	LYP2-P	(52)
	Acetylation modification	Acetic anhydride method - Acetylation modification is the addition of acetyl groups to the branched chains of polysaccharides, so that the branched chains of polysaccharides are fully unfolded, exposing more sugar hydroxyl or carboxyl groups inherent in the sugar, thus improving its water solubility and more conducive to activity	The pH of the reaction system was always maintained in the range of 8.0–10.0 by adding sodium hydroxide solution and acetic anhydride alternately under certain temperature conditions until the acetic anhydride was added, and the reaction was adjusted to neutral with hydrochloric acid solution after stirring for a period of time. Finally, dialysis, concentration, alcohol precipitation and freeze-drying were performed.	Hydroxylamine colorimetric assay	Ac-LYP2	(53)

#### Sulfated modification

Among the chemical modification methods, sulfation is widely used in molecular modification of polysaccharides (54), because it not only increases the solubility of polysaccharides, but also provides and creates new physiological functions. Specifically, the principle is to add sulfuric acid groups to the sugar groups of polysaccharides to change the structure of polysaccharides, thus increasing their biological activities, such as immunomodulatory (55), anti-tumor (56), anti-oxidant (57), hypoglycemic (58) and anti-viral (54) activities. After sulfation modification, the chemical composition, Mw and monosaccharide composition of LPs were changed, and the activities of anti-glycosylation and anti-coagulant were also significantly enhanced (59). The sulfated derivative of LPs (LP1-S) was prepared by sulfation by Jiang et al., and the degree of substitution (DS) was determined to be 2.011. Two characteristic absorption bands (1223 and 640 cm^−1^) appeared in FT-IR spectrum of LP1-S, indicating that the sulfation reaction did take place, the structural characteristics are shown in Table 1. Preliminary in *vitro* experiments demonstrated that LP1-S exhibits higher anti-proliferative activity than LP1 against human nasopharyngeal carcinoma (HONE1) cells, which may be due to the sulfate moiety in its structure. Therefore, these results suggested that LP1-S may be more useful than LP1 for the development of safe antitumor drugs or health foods (30). In addition, several studies have shown that the biological activity of sulfated LPs is mainly reflected in the sulfate groups. The number and location of the sulfate groups were different, and the biological activities were also different. Generally, the higher the degree of sulfation, the stronger the biological activity, which affects the degree of sulfation substitution. Huang et al. experimentally determined that the factors affecting the degree of sulfation substitution are: reaction time > reaction temperature > amount of reagent addition (47). The modification of LPs by sulfation aimed to produce sulfated LPs with low cost, high product volume and high biological activity (60).

#### Carboxymethylated modification

Carboxymethylation refers to the introduction of carboxymethyl groups on the branched chains of polysaccharides (61). Studies have shown that the introduction of carboxymethyl groups enhances the water solubility of LPs, and even the degree of substitution (DS) of carboxymethylation is proportional to its water solubility, which is one of the key factors to promote its active role (62). Wei et al. used RSM to optimize the preparation process of carboxymethylated LPs (CM-LYP). The response surface analysis was performed by single-factor test on the concentration of monochloroacetic acid (MCA), reaction temperature and reaction time with the degree of carboxymethyl substitution as a measure, and a regression model between the degree of carboxymethyl substitution and each influencing factor was established. They finally obtained the optimal process conditions for the preparation of CM-LYP as MCA concentration of 1.2 mol/L, reaction temperature of 73°C, and reaction time of 3.2 h, at which the carboxymethyl substitution degree of LPs was as high as 1.053. Meanwhile, the results of antioxidant activity experiments pointed out that at the same dose concentration (3200 μg/mL), the scavenging rate of hydroxyl radicals and superoxide anion radicals as well as the inhibition of lipid peroxidation and H_2_O_2_-induced erythrocyte hemolysis were stronger with CM-LYP than with LYP before modification. The results of *in vivo* immunoreactivity assay also pointed out that CM-LYP has better immunoreactivity than LYP in terms of increasing spleen index, promoting serum hemolysin formation, and increasing lysozyme content in serum and spleen of immunosuppressed mice. This indicated to some extent that the introduction of carboxymethyl is beneficial to improve the immunomodulatory and antioxidant activities of LYP (49).

#### Enzymatic modification

We stated earlier that enzyme technology can be used to assist in the extraction of LPs, and in addition to that, enzymes can also be used to modify the structure of LPs. Compared with chemical modifications, enzymatic modification has the advantages of high specificity and high efficiency. After modification by enzymatic degradation, the Mw of LPs was reduced, their relative molecular masses were relatively uniform, and the process had little effect on the backbone of LPs (63). The aim of enzymatic modification was to utilize the specificity and selectivity of enzymes to specifically modify the structure of LPs to make better produce biopolymers with ameliorative mechanical and/or biological functional properties (64). Modern research has found that polysaccharides, when present in the form of oligosaccharides (defined as compounds containing 2–10 glycosidic bonds polymerized), act as prebiotics. Consuming these oligosaccharides can be used to modulate the intestinal microbiota, helping to create a healthy intestinal ecology and improve mineral absorption in the colon. Cheng et al. used a commercial enzyme, Viscozyme L, from *Aspergillus aculeatus*, a mixture containing pectinase, glucanase, cellulase and Fructo-furanosidase (FFase), in juicing longan fruit pulp. The purpose of using it was to increase the juice yield and clarity based on pectinase, glucanase and cellulase, and then converted the sucrose in it to Fructo-oligosaccharide by transfructosylation of FFase. They determined the content of water-soluble LPs on the basis of colorimetric method using phenol-sulfuric acid colorimetric method. The experimental results showed that the content of water-soluble LPs such as pectin increased significantly after enzyme treatment, while the sucrose content decreased significantly, and at the same time, a lot of fructo-oligosaccharides, including 1-kestose and nystose, were synthesized. In addition, the results of High-Performance Gel Permeation Chromatography (GPS) experiments showed that the content of fraction 1, representing the largest size of LPs, was significantly decreased after enzyme treatment, while the content of smaller size fractions 3 and 5 was increased, indicating that large polysaccharides could be hydrolyzed into small molecules by enzymatic digestion. Afterwards, they investigated the *in vitro* stimulation of *Lactobacillus* by LPs obtained before and after enzyme treatment. The experimental results pointed out that the stimulation of *Streptococcus thermophilus, Lactobacillus acidophilus* and *Lactobacillus delbrueckii* reference strains by LPs and Fos in longan juice was significantly improved after enzyme treatment (65). In conclusion, the content and molecular size distribution of LPs in longan juice changed significantly after enzyme treatment, implying that enzymes modify the structure of LPs and thus affect the expression of their biological activities. These showed the good potential of enzyme-treated LPs as functional foods, nutraceuticals and dietary prebiotics (50).

#### Maillard reaction modification

Maillard reaction (MR) is based on the carbonyl ammonia reaction between the carbonyl group of reducing sugar and the amino group of amino compounds (66), which involves a series of reactions such as condensation, degradation, cracking and polymerization (67), ultimately producing complex compounds such as ketones, pyrans and pyridines (68). MR also known as “non-enzymatic browning reaction,” is a natural, non-toxic and widely available non-enzymatic browning in the food industry that allows effective macromolecular modification under mild and safe conditions and without additional chemicals (29, 69). Modern studies have shown that some amino acids, such as lysine (Lys), proline (Pro) or glycine (Gly), among others, are present in the samples obtained after the extraction and purification of LPs. Under certain conditions, they might form an MR system with LPs, whereby trigger MR (37). The changes in browning degree and Mw distribution can indicate the binding state of LPs and amino acids (70). For example, Yi et al. found that the reaction of LPs and Lys at pH = 9 and T = 100 °C for 4 h resulted in MR, which could yield the product MLPs. There were significant differences in the physicochemical properties of MLPs compared to unreacted LPs. This was demonstrated by the significantly higher browning of the MLPs and the fact that 6% of the LPs had a component Mw >7.07 kDa, while 45% of the MLPs had a component Mw >264.1 kDa. Then they also investigated the chemical composition and structural characteristics of MLPs and found that the formation of MLPs from LPs and Lys by MR may be due to the fact that the tight conformation of LPs dissociates in alkaline and high temperature environments and the lysine molecule binds to the carbonyl group of LPs mainly through a free amino group (29). In addition, the results of bioactivity studies showed that MLPs have stronger free radical scavenging and immunostimulatory effects on macrophages compared to LPs, but weaker growth inhibition of cancer cells. These changes are related to lysine modifications and moreover to complex conformational relationships.

Secondly, MR between LPs and proteins is also a major feature of MR modification. The structural features of LPs-protein complexes can be monitored by measuring their free amino content, ultraviolet-visible spectrum (UV-Vis), FT-IR and Mw distribution to determine whether a MR occurs between LPs and proteins (71). Furthermore, *In vitro* activity evaluation showed that dry-heat-induced MR effectively enhances the anti-oxidant, anti-tumor and immune-stimulating activities of LPs (72). Meanwhile, the LPs-protein polymers formed by MR usually exhibit better properties, such as emulsification, thermal stability and solubility, than pure proteins or simple mixtures of protein-LPs. Actually, substances containing polysaccharides and proteins can undergo MR under heating conditions, which can be used for further processing and storage of these substances. Longan fruit pulp contains a large number of polysaccharides and protein, and it has a short storage period due to its high perishability and susceptibility to post-harvest diseases (73). Therefore, the methods of heating and drying are often utilized to store longan fruit pulp. During the heat process, there is a MR (also known as glycosylation reaction) between longan fruit pulp protein and LPs (74). LPs are linked to protein through the covalent bond between the free amino group of amino acids (mainly lysine and arginine) and the carbonyl groups of LPs (69). Therefore, the LP-protein Maillard modification is a good method to promote the biological activity of LPs and better storage of longan fruit pulp, but the relationship between its effect and the reaction stage remains to be further studied (37).

In conclusion, the MR reaction not only promoted changes in the primary structure of LPs but also triggered a series of changes in their higher-level structures, and these changes proved that MR is a promising method for modifying natural polysaccharides to obtain better biological properties. Therefore, MR-based probing of the effects of binding amino compounds on the structure and bioactivity of LPs can help to understand the structure-activity relationship of LPs (69).

#### Microbial fermentation modification

Chemical modification can change the structure of LPs by introducing substituents to affect their biological activities. However, chemical modification generally requires the use of organic solvents, strong acids or strong bases, which to some extent limits its application in practical production. Simultaneously, other modification methods, such as microbial biotransformation and enzyme modification, have attracted more and more attention due to their advantages of high efficiency, high specificity and products containing less harmful substances. Notably, fermentation using Lactic acid bacteria is an important fruit and vegetable processing technique that has been reused in recent years because of its ability to maintain and/or enhance the safety, nutrition, sensory and shelf-life characteristics of fruits and vegetables (75, 76). Recently, researches have applied fermentation technology to the structural modification of plant polysaccharides to make better physicochemical properties and biological activity of polysaccharides (77, 78). For example, Huang et al. applied *Lactobacillus fermentum* without producing extracellular polysaccharides to ferment longan fruit pulp juice at 37°C for 24 h, and then extracted LPs with hot water. The LPs isolated from unfermented and fermented longan fruit pulp juice were named as LP and LP-F. After structure analysis, it was found that LP-F has lower Mw, and contains less Glc, neutral sugar and uronic acid, but contains more Gal, Man, Ara and Rha. This revealed to some extent that LP and LP-F have some structural differences (28). In addition, they compared the physicochemical and prebiotics properties of LP-F extracted at different fermentation times and found that at different fermentation time points, the yield of LP-F, monosaccharide composition, Mw, content of neutral sugar and uronic acid changed obviously. The structural characteristics are shown in Table 1. After fermented by lactic acid bacteria, the structures of LPs were modified by lactic acid bacteria, and the prebiotic activity was improved accordingly. Among the tested fermented LPs, LP-12 showed the strongest prebiotic activity (41). The results of the fermentation experiments also showed that LPs are fermented by the intestinal microbiota after entering the human body for 48 h. The LP-F fermentation cultures contained higher levels of total short-chain fatty acids (SCFAs) and acetic acid, followed by higher numbers of *Enterococcus, Bifidobacterium* and *Clostridium* than LP fermentation cultures, but LP-F fermentation cultures showed lower pH and lower carbohydrate percentages than LP. Thus, LP-F was more susceptible to fermentation by the human intestinal microbiota than LP. This effect might also be attributed to the special chemical properties and structure of LP-F, including good solubility, low apparent viscosity, particle size, Mw, and different composition of monosaccharides (79).

#### Others

In addition to the structural modification methods above, there was some other methods also involved in some literatures. For example, alkali dissociation was one of the traditional methods to prepare modified LPs. In the study, longan fruit pulp polysaccharides (LPI) were treated with sodium hydroxide, the alkali solution first cleaved the glycosidic bond of LPI, and degraded LPI into fragments with low Mw. Thus, alkali dissociation depolymerized LPI with tight and high tissue conformation to obtain its derivatives whose solubility and biological activity were strengthened (51). Similar to sulfation modification, phosphorylation modification involved the introduction of phosphate groups into LPs. After modification, the stability of LPs was improved and the anti-genic properties of LPs in the body could be maintained. Studies had shown that longan fruit pulp polysaccharide (LYP2) is phosphorylated and modified. Phosphorylated-LYP2 had a triple helix structure, and the triple helix structure was a characteristic structure of polysaccharides with anti-tumor activity (52). There was also a small amount of literature mentioning acetylated modified LPs (53). In short, molecular modification changed the size structure, Mw, and the type, number and position of substituents of LPs, thereby affecting its biological activity.

## Biological activities

It has been documented that both natural and modified polysaccharides usually exhibit a variety of biological activities. The Mw and chemical structure of polysaccharides are two main factors that determine the biological activities. Modern pharmacological activity studies have shown that LPs have immunomodulatory, anti-tumor, anti-oxidation, anti-glycosylation and hepatoprotective activities. Generally, diversified extraction and purification technologies, unequal treatment and application methods and different research techniques will affect the biological activities of LPs. Biological activity testing and research of LPs are mainly based on longan fruit pulp crude polysaccharides, which also brings obstacles to prove the exact active substances. Therefore, to promote the product development and functional application of LPs, we systematically reviewed the biological activities of LPs to provide a comprehensive reference for the basic research of LPs.

### Immunoregulatory activity

Immunomodulatory activity is considered to be one of the important strategies to ameliorate the overall defense mechanisms in normal individuals and cancer patients. Experimental evidence *in vivo* and *in vitro* suggests that polysaccharides can exhibit immunomodulatory functions by stimulating cellular and humoral immune responses. In the meantime, the immunomodulatory function of polysaccharides might start from activating effector cells, such as natural killer (NK) cells, lymphocytes and macrophages, and the proliferation of spleen cells and the enhanced phagocytosis of macrophages were also indicators of enhanced immune regulation (80). Several recent reports have shown that LPs have certain immunomodulatory activities *in vivo* and *in vitro*, and their immunomodulatory mechanisms are diverse. However, the variety of LPs in these reports suggests that there may be multiple LPs involved in immunomodulation (44). Therefore, to better revealed the immunomodulatory activity mechanism of LPs and their structure-activity relationships, modern studies had characterized the structures of LPs by GC-MS, FT-IR and NMR *etc*., and evaluated the immunomodulatory activities of LPs by relevant experiments.

**Figure 4:**
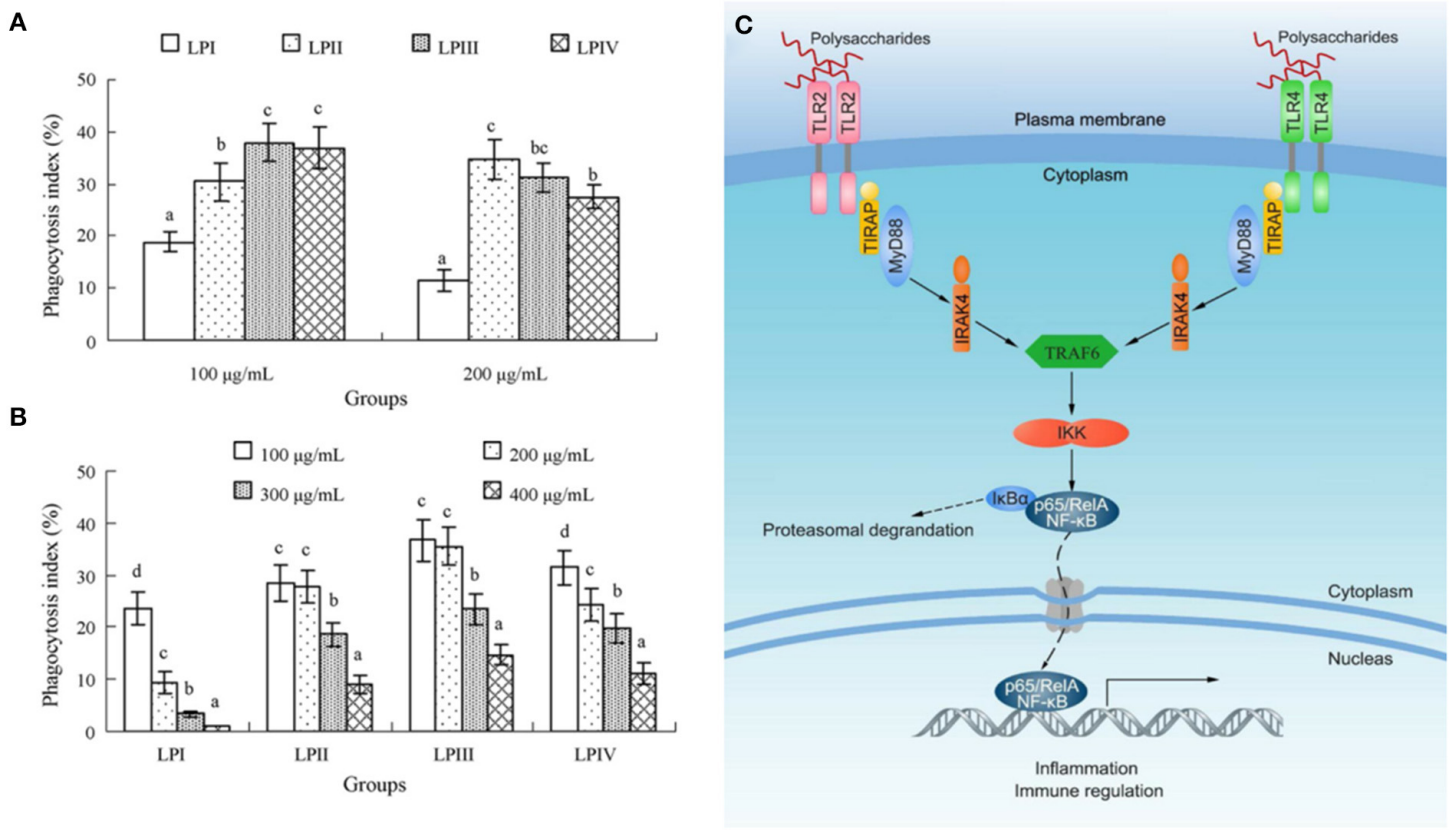
The activation of macrophages and the enhancement of phagocytic function are indicators of the enhancement of LPs immunomodulation, anti-tumor and anti-inflammatory activities. **(A)** The comparison of phagocytosis indexes of LPI–IV at 100 or 200 μg/mL. **(B)** The phagocytosis indexes of LPI-IV in the dose range of 100–400 μg/mL (34). **(C)** Possible molecular mechanism of LPs activating macrophages (35).

First, it was noted that LPs stimulate the proliferation of splenic lymphocytes and phagocytosis of macrophages (81). And among them, macrophages play an extremely critical role in innate and acquired immune regulation (82). In general, delayed-type hypersensitivity (DTH) was considered to be the diagnostic expression of related immune responses. The medium-dose (200 mg/kg) and low-dose (100 mg/kg) LPs extracted by ultrasound showed strong immunoregulatory effects on mice model. Compared with the model control group, it could significantly enhance the DTH response, macrophage phagocytic function and concanavalin A (ConA)-stimulated spleen cell proliferation in mice (83). Min et al. prepared longan fruit pulp homogenous polysaccharide-protein complexes (LPP) by alkali extraction and acid precipitation method, it enhanced the immune function of mice through several ways, such as enhanced the production of anti-chicken red blood cell anti-body and phagocytosis of macrophage, promoted the proliferation of ConA-induced splenocyte and the secretion of cytokines in serum (33). Secondly, based on this, Yi et al. obtained LPIIa from longan fruit pulp and investigated its physicochemical properties and immune-stimulating effects on macrophages. Studies have shown that in the dose range of 100–400 μg/mL, LPIIa enhances the nitric oxide (NO) production and macrophage phagocytosis function. Moreover, at a dose of 200 μg/mL, LPIIa significantly increased the activity of macrophage inducible nitric oxide synthase (iNOS), and the secretion of interleukin-6 (IL-6) and tumor necrosis factor-α (TNF-α). However, these effects were significantly reduced after Toll-like receptor 4 (TLR4) or TLR2 was blocked. Similarly, specific inhibitors such as p38 mitogen-activated protein kinase (MAPK), protein kinase C (PKC), phosphatidyl inositol 3-kinase and nuclear factor-κB (NF-κB) also selectively inhibited LPIIa immune-stimulating activity against macrophages. These results suggested that its immunostimulatory signal may be mediated by TLR4 and TLR2, and subsequently activate the p38MAPK and NF-κB pathways. Therefore, LPIIa had strong immunomodulatory activity by stimulating macrophages and could be used as an adjuvant for immunotherapy (39). Besides, Yi et al. obtained four fractions (LPI-IV) from the LPs water extract by DEAE anion exchange column chromatography, and evaluated the immunomodulatory activity of these polysaccharides *in vitro*. They concluded that compared with normal control group, except for LPI, the other three polysaccharides significantly stimulate lymphocyte proliferation within the dose range of 100–400 μg/mL, and also selectively stimulate B cells but not T cells. Furthermore, their promoting effects on lipopolysaccharide (LPS) -induced cell proliferation and their inhibitory effects on ConA-induced cell proliferation were LPIII > LPIV > LPII > LPI, respectively. The optimal dose of all components was 100 μg/mL, and all of them could enhance the phagocytosis of macrophages. Among them, LPII as a potential immunoadjuvant had high yield and activity (34). In addition, they also studied the relationship between molecular conformation and immunomodulatory activity of LPs. LPI had a spherical and rigid rod-like conformation, had no immune stimulation on NK and splenocytes cells in the dose range of 100–400 μg/mL. LPI1 or LPI2, as the alkali dissociative derivative of LPI, significantly enhanced the proliferation of splenocyte and cytotoxicity of NK cells due to the existence of slightly dissociated spherical conformation or single-helix chain. However, its contribution to macrophage phagocytosis was weak (51). Rong et al. investigated the immunomodulatory activity of LPs by comparing the effects of polysaccharides on the phagocytosis of macrophages. They isolated an active polysaccharide (LPD2) from longan fruit pulp and studied its structure and activity. Immune activity tests showed that LPD2 significantly promotes the phagocytic function and proliferation of lymphocytes, and promotes the secretion of IL-6 and NO by macrophages, suggesting that LPD2 can effectively induce the functional activation of macrophages. Meanwhile, LPD2 had a strong stimulating effect on the proliferation of splenic lymphocytes, which are important participants in innate immunity and adaptive immunity, suggesting that LPD2 has a significant impact on the activation of immune function in the body (Figure 4) (35). In addition, LPs also mediated immune-stimulating activity by interacting with various receptors and/or regulating intracellular signaling pathways after binding to various receptors, such as TLR4 (84), the lectin receptor of dectin-1, and complement receptor (85), *etc*. In short, the immunostimulatory activity of LPs might be due to direct or indirect interaction with immune system components, triggering different cellular and molecular events. The direct stimulation of immune cells such as macrophages, lymphocytes, neutrophils, monocytes, dendritic cells and complement proteins involved specific recognition receptors, which determine the final response (86).

**Figure 5:**
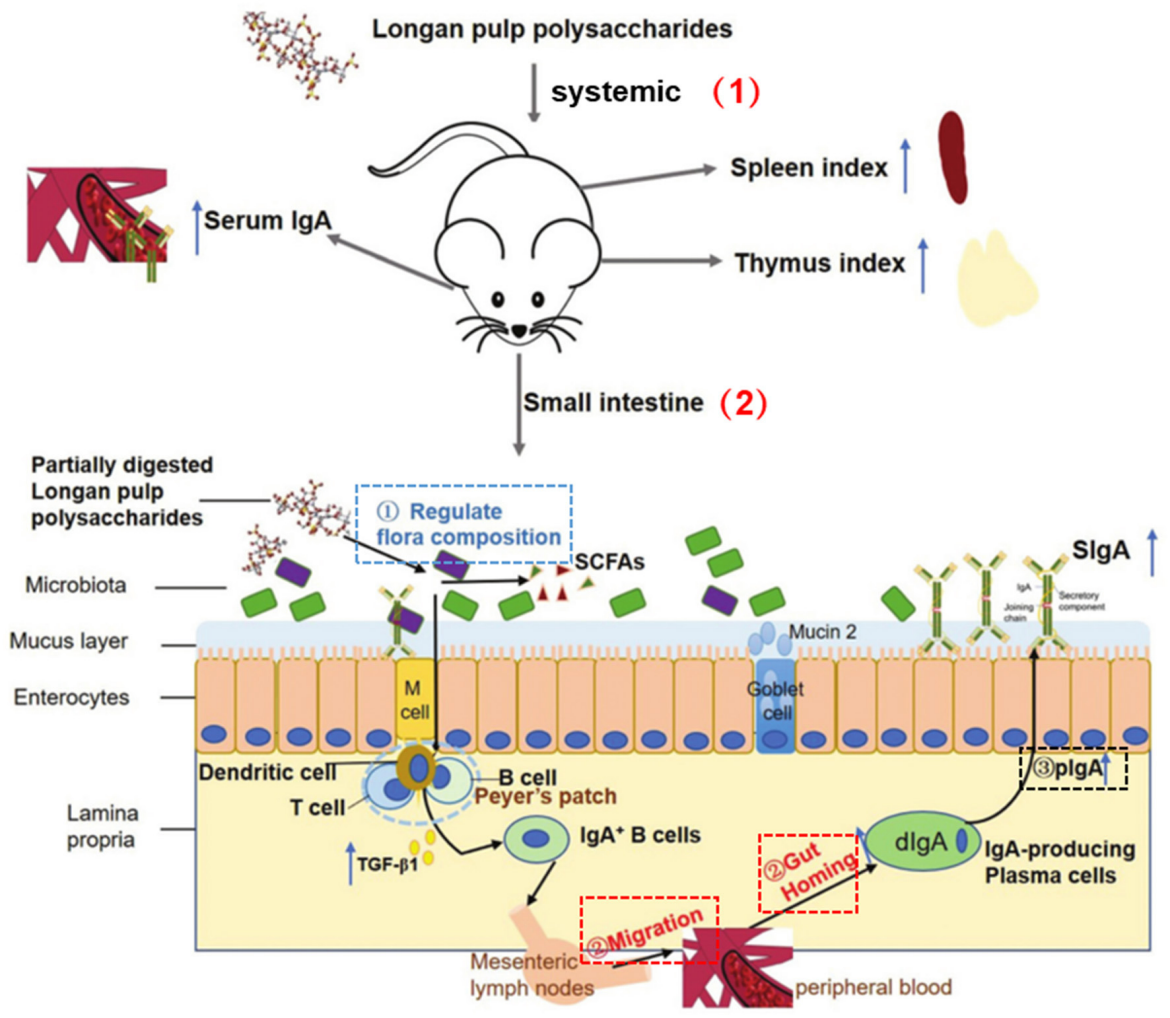
LP regulated systemic and intestinal immunity (89). (1) Systemic immunity: LP increased spleen and thymus indexes and serum IgA level. (2) Intestinal immunity: LP regulated intestinal microbiota composition *via* utilization by partial commensal bacteria; LP promoted the migration and gut homing of IgA+ plasma cells; LP directly stimulated transcytosis of dIgA-pIgR and pIgR.

It is worth mentioning that LPs can also exert immunomodulatory activity by acting on the intestine. Intestinal tract is the largest immune organ in the host, proper intestinal immune response of the steady state as well as the health of the host play an important role in maintaining intestinal barrier (87). The Mw of LPs is relatively large, generally more than 104 Da, so they are difficult to be absorbed directly through the gut. However, studies have displayed that the host indigestible polysaccharides are the most important food source of intestinal flora, so a diet rich in these polysaccharides was crucial for maintaining a healthy gut (88). Zhang et al. studied the effects of LPs on mice gut microbiota and its interaction with the host immune system using a multi-omics method. The results noted that LPs significantly increase the typical intestinal immune indexes of mice, and the changes caused by LPs in the microbiota are related to the immunomodulatory activity (44). In addition, there were studies aiming to investigate the effects of LPs on the systemic immunity and intestinal mucosal immunity of immunosuppressed mice by observing the synthesis and secretion of intestinal secretory immunoglobulin A (SIgA). And the results showed that the LPs improve cyclophosphamide (CTX)-treated in mice thymus and spleen indexes. The level of serum immunoglobulin A (IgA) and the secretion of SIgA in the intestinal lumen were also increased by the action of LPs. Therefore, appropriate consumption of LPs helped to strengthen the mucosal barrier by promoting the secretion of intestinal SIgA, thereby enhancing systemic immunity and intestinal mucosal immunity (Figure 5) (89).

It is also noteworthy that the structurally modified LPs have greater immunomodulatory activity. For example, Yi et al. studied the immunomodulatory effects of LP-protein complex (LP3) in immunosuppressed mice models. Oral administration of 100 mg·kg^−1^·d^−1^ LP3 could stimulate macrophage/lymphocyte activation and anti-body/cytokine secretion, and could also enhance splenocyte proliferation, anti-body production, cytokine secretion, macrophage phagocytosis and NK cytotoxicity in immunosuppressed mice. However, the structural characteristics of LP3 and the structural/immunomodulatory relationship need to be further studied (32). Jiang et al. indicated that all experimental doses (25–100 μg/mL) of sulfated LP1 (LP1-S) and LP1 significantly increased the proliferation of lymphocyte cells, spleen cells and LPS-induced proliferation of spleen cells, but LP1-S showed higher immunomodulatory activity than LP1. It might be caused by the sulfate group in the structure of LP1-S (30). Phosphorylation and acid hydrolysis were also used to modify LPs. Compared with unmodified LPs, their derivatives showed stronger effects in enhancing macrophage phagocytosis, stimulating lymphocyte proliferation and regulating cytokines, *etc*. (28). Therefore, to understand the structural characteristics of longan fruit pulp active polysaccharide was helpful to understand the application mechanism of LPs as an immune regulator.

### Anti-tumor activity

According to the antitumor mechanism of polysaccharides, the antineoplastic activity of polysaccharides can be divided into two types: (1) polysaccharides inhibit the proliferation of cancer cells by activating the immune system of the body; (2) polysaccharides directly inhibit the proliferation of tumor cells (90). At present, the mortality rate of tumor-related diseases is quite high, and the effectiveness of cancer treatment is limited. Many anti-cancer drugs are known to be immunosuppressants, which inhibit tumor growth and impair the immune system. Therefore, the discovery and identification of new anti-tumor drugs that can play an immunomodulatory role has become an important target of immunopharmacology and oncology therapy (91). Zhong et al. noted that UELP (ultrasound-extracted LPs) has a good anti-tumor effect on S180 tumors *in vivo*. Importantly, the experimental dose (200 mg/kg) of UELP had extreme anti-tumor effects, with a maximum inhibition rate of nearly 85% (83). At the same time, LPs also had anti-tumor effects in S180 tumor mice, which may be shown by the immunomodulatory mechanism as an immune adjuvant for the treatment of cancer. However, their direct inhibitory effect on tumor cells was not available (43). Meng et al. extracted and successfully purified a new WPS (LP1) from longan fruit pulp. *In vitro* experiments had shown that LP1 has significant anti-tumor activity against HO8910 and SKOV3 tumor cells, with inhibition rates of 50 and 40%, respectively. Meanwhile, LP1 was observed to prevent the development of tumor cells by directly killing tumor cells *in vitro* and in mice and improving immune activity. Therefore, LP1 has significant anti-tumor potential and can be used as a safe and effective reagent to treat corresponding diseases (24). LPs could also directly inhibit the proliferation of tumor cells cultured *in vitro*. Yi et al. obtained three kinds of LPs with different structures through different extraction methods, named LPI, LPII and LPIII. Anti-tumor studies had shown that in the range of 50–400 μg/mL, those three LPs showed direct inhibitory effects on HepG2, A549 and HeLa cells in a positive dose-dependent manner, and the inhibition rate of 400 μg/mL was significantly higher than other doses. In addition, the activity of 400 μg/mL LPIII, especially its inhibitory effect on the proliferation of hepatoma, was stronger than other activities, which may be related to its flexible conformation (92). The anti-tumor activity of LPs was also improved after modification by structural modification. Jiang et al. used purified longan fruit pulp WSP (LP1) as the raw material to prepare a sulfated derivative (LP1-S) by sulfuric acid method, and the measured DS was 2.011. Studies had shown that LP1-S has stronger anti-tumor activity compared with LP1, which may be related to its sulfate content (46). Anti-cancer tests showed that LP1-S has a certain inhibitory effect on the growth of hepatoma cells. However, it did not show any cytotoxicity against MCF-7 cells. In conclusion, LPs can be used as a potential anti-tumor adjuvant in food and drug therapy, but its anti-tumor activity and structure-activity relationship *in vivo* need further study (30).

### Prebiotic activity

Current literatures have shown that the prebiotics activity LPs mainly related to its influence on gut microbiota. The adjustment of the microbiota can ameliorate insulin resistance, blood glucose and glucose tolerance control and reduce inflammation (93), so LPs can be used as prebiotics and become a potential treatment tool for the treatment and prevention of obesity (94). Besides, LPs can selectively benefit a number of living microbial species called prebiotics in the human gut, and exert its health benefits by boosting the number of prebiotics in the gut and regulating its metabolites (95, 96). Therefore, evaluating the prebiotic activity of LPs is crucial to evaluate the possible health effects of LPs. Huang et al. showed that LP-SE has stronger prebiotic activity than LP-H and LP-S. Firstly, LP-SE had a higher extraction efficiency than LP-H and LP-S, so it had a higher sugar content and a lower Mw. LP-SE was considered to have a better proliferation effect. Secondly, the monosaccharide composition and glycosidic bond of LPs affected the prebiotic activity. Studies have shown that LP-SE has better prebiotic activity, which may be due to the fact that it contains more Ara and Gal, as well as the specific glycosidic bond → 5)-α*-L*-Ara*f*-(1 → . Moreover, the viscosity and solubility of LPs were also one of the important factors affecting the prebiotics activity. LP-SE with good water solubility and low viscosity was more likely to be quickly and completely utilized by prebiotics, so it showed stronger prebiotics activity than LP-H and LP-S (22). Furthermore, the prebiotic activity of enzyme-modified LPs was improved to some extent. Cheng et al. investigated the use of commercial Viscozyme L in the obtained longan fruit pulp juice, which has the potential to convert sucrose into fructo-oligosaccharides. The results showed that enzyme treatment significantly changes the Mw distribution and monosaccharide composition of WPS. In addition, the enzyme-treated longan fruit pulp juice and its ethanol-soluble polysaccharide components enhanced the stimulating effect on the growth of intestinal probiotics, such as the growth of *Lactobacillus delbrueckii, Streptococcus thermophiles* and *Lactobacillus acidophilus*. All of them indicated that the oligo-polysaccharides contained in the enzyme-treated longan fruit pulp juice have better prebiotics activity (50).

### Anti-oxidant activity

The anti-oxidant activity of polysaccharides is mainly demonstrated by the direct scavenging effect on hydroxyl radical (∙OH), superoxide anion radical (∙O^2−^) and hydrogen peroxide (H_2_0_2_) (97). Free radicals are produced by aerobic reactions in the body. They poison the body in different ways and are closely related to the occurrence of multiple diseases (98, 99). When Zhong et al. investigated the radical scavenging activity of UELP, they found that experimental concentrations (5–35 mg/mL) of UELP have excellent scavenging activity for ∙OH and ∙DPPH, and both scavenging rates were significantly enhanced with increasing UELP concentrations, and a larger dose (35 mg/mL) of UELP showed almost complete scavenging effect (83). Liu et al. obtained longan wine solution by fermenting longan fruit pulp juice with *Saccharomyces cerevisiae*. Longan fruit pulp wine polysaccharide (LWP), the main functional component of longan fruit pulp wine, was separated into 4 fragments with different Mw by ultrafiltration system. It was showed that with the increase of Mw, the ∙DPPH scavenging rate of longan fruit pulp wine and four LP sections was gradually increased. Compared with longan fruit pulp wine, LWP with a Mw of 10–30 kDa or a <3 kDa had significant anti-oxidant activity in the volume range of 0.2–2.0 mL. When the sample volume was >1.6 mL, the 10–30 kDa LWP had the highest ∙DPPH scavenging rate (100). It is well-known that oxidative stress caused by free radicals and/or reactive oxygen species can cause organ damage. Chen et al. studied focal cerebral ischemia/reperfusion injury and its mechanism. The results showed that compared with the ischemia/reperfusion group, the LPs can significantly reduce the nervous system score, the cerebral infarction volume, the brain water content, malondialdehyde (MDA) content, myeloperoxidase (MPO) activity, TNF-α and IL-1β levels, Bax expression, and increase superoxide dismutase (SOD), glutathione (GSH), glutathione peroxidase (GSH-Px) activity and expression of Bcl-2. Therefore, LPs could be used as an effective polysaccharide with significant protective effect on cerebral ischemia/reperfusion injury, which may be related to the mechanism of reducing oxidative stress *in vivo* and *in vitro* (101). In addition, compared with unmodified LPs, MLPs, acetylated and carboxymethylated LPs derivatives had stronger anti-oxidant activity (102).

### Anti-inflammatory activity

Inflammation is a defense response of the body to stimulation, which is characterized by redness, swelling, heat, pain and dysfunction. Chronic inflammations and disorders may lead to secondary injury and immunopathology in the host (103). LPIIa was purified from longan fruit pulp, and a co-culture model of Caco-2 cells and Raw264.7 macrophages was used to study its anti-inflammatory activity and intestinal barrier protection. The results showed that LPIIa inhibits the production of inflammatory mediators, including TNF-α, IL-6, NO (104) and PGE2, and reduces the expression of iNOS and cyclooxygenase-2 (COX-2) genes in LPS-induced Raw264.7 macrophages. In addition, LPIIa also decreased the expression of the intestinal tight junction channel protein Claudin-2 and increased the expression of the tight junction barrier protein ZO-1 in Caco-2 cells. These results indicated that LPIIa has a certain therapeutic effect on inflammatory bowel disease (IBD) (31).

### Inhibition of AChE activity

Longan fruit pulp has been reported to have beneficial effects on memory in humans and is often used in Chinese medicine to treat amnesia, yet the mechanism by which longan fruit pulp affects memory function is unclear. Some studies have pointed out that acetylcholine plays an important role in memory function in the brain, and a large body of evidence supports the mechanism by which choline has the ability to regulate learning and memory function. And increasing acetylcholine levels with acetylcholinesterase (AChE) inhibitors is currently one of the most effective strategies for the treatment of Alzheimer's disease (105). Bai et al. compared the AChE inhibitory activity of LPs obtained by different extraction methods. All LPs showed moderate and concentration-dependent inhibition of AChE at concentrations ranging from 0.1 to 3.0 mg/mL. At a concentration of 3.0 mg/mL, the AChE inhibitory activities of LP-H, LP-E, LP-U and LP-UE were 18.15%, 19.31%, 20.17% and 25.40%, respectively. These results indicated that EAE and UHP-assisted extraction can improve the biological activity of LPs. Compared with the other three types of LPs, LP-UE had the highest AChE inhibitory activity, suggesting that LP-UE may have the potential to meliorate cognitive dysfunction. Thus, LP-UE may be a potential AChE inhibitor, and the regulation of the cholinergic system may be one of the pharmacological mechanisms of LPs to ameliorate memory impairment (3).

### Others

In addition to the above activities, LPs also has some biological activities in intestinal protection, promoting cartilage growth and other aspects. Studies have shown that early intake of LPs can reduce the intestinal mucosal damage caused by chemotherapy by increasing the expression of mucin, adherence junction (AJ) and tight junction (TJ) complexes, thereby promoting the renewal of the intestinal mucosal and the morphological integrity of the intestine *in vivo*, so as to achieve the protective effect of LPs on the intestine (36). In addition, the effects of LPs on rabbit articular chondrocytes were explored by detecting the activity, morphology, cartilage-specific gene expression and glycosaminoglycan synthesis. The results showed that, similar to the positive group using TGF-β growth factor, LPs effectively promote the growth of chondrocytes and enhance the secretion and synthesis of cartilage extracellular matrix by up-regulating the expression levels of sox9, aggrecan and collagen II. Compared with the negative control group, the expression of type I collagen gene was down-regulated, indicating that LPs inhibit chondrocyte dedifferentiation. All these suggested that LPs can replace growth factor in the treatment of autologous chondrocyte implantation (ACI), which lays the foundation for the development of new drugs for the treatment of articular cartilage defects (106).

## Patent registration of LPs

**Figure 6:**
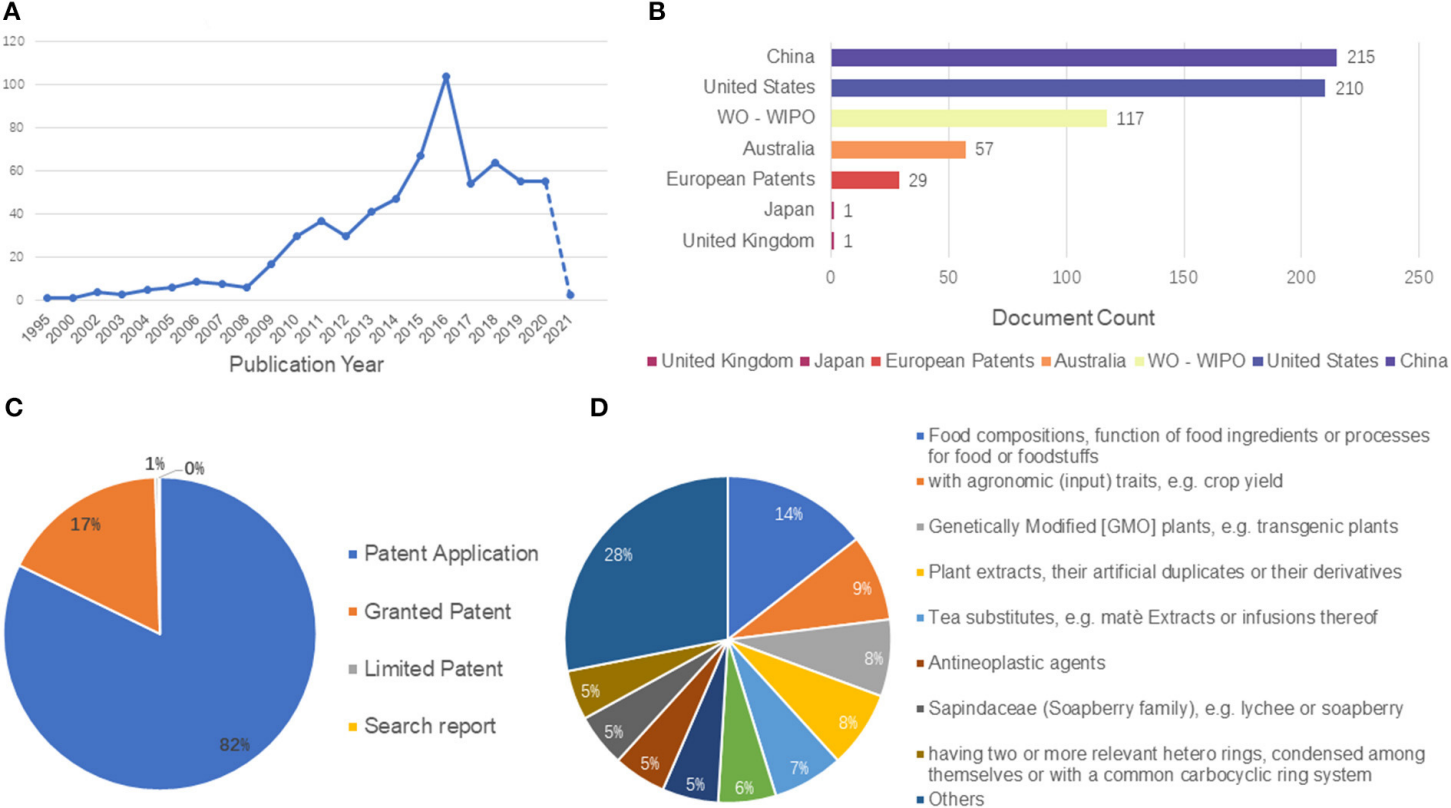
The analysis of 630 patents browsed was obtained by Lens.org using the search term “Longan polysaccharide.” **(A)** Patent applications per years, **(B)** Document type, **(C)** Jurisdictions, and **(D)** CPC Classification.

Currently, there are 630 patents related to LPs worldwide, of which 82.2% are patent applications, 17.3% are granted patents and <1% are limited patents. Among them, the jurisdiction of these patents is mainly distributed in China, the United States of America, Would Intellectual Property Organization (WO-WIPO), Europe and Australia. According to the relevant data, China and the United States have the largest number of patents, accounting for 67% of the total. In these patents, LPs are mainly used as food or food processing products, health products and dietary supplements. Some studies have specified that LPs and other substances together to make powder preparations with enhancing immune function broadens the utilization of LPs as a kind of health food. In addition, some patents also introduced that LPs can be used to make solid drinks to regulate Qi deficiency (a TCM term describing “chronic fatigue syndrome”) constitution, powder with enhancing immunity, herbal composition to treat nervous system diseases and improve memory damage, pharmaceutical composition to treat cardiovascular and cerebrovascular diseases and as hair growth agent, *etc*. Their preparation methods and applications were also mentioned in turn. All these provided a theoretical basis for the patent development of LPs. In a word, although many scholars have made great contributions to the research of LPs in terms of basic research, the commercial and industrial development of LPs products is still in the initial stage, and there are still some difficulties in the development of most LP-related products in the actual industrial production. The analysis of 630 patents browsed was obtained by Lens.org using the search term “Longan polysaccharide,” the patent details of LPs are shown in Figure 6.

## Conclusions and perspectives

Longan fruit pulp, as one of the representative substances of medicinal and food homology, has attracted more and more attention from researchers because of its high edible and adjuvant therapeutic value. LPs, as one of the main biologically active components in longan fruit pulp, has been proven to be able to obtain homogeneous LPs with different structural characteristics from longan fruit pulp through different extraction and purification methods, and they also exhibit different biological activities. In recent years, researchers have conducted several studies on the structure and bioactivity of LPs, which focused on the traditional HWE, chemical composition, structure, immunostimulatory and antioxidant activities of LPs. Meanwhile, several more efficient methods have been applied in the extraction of LPs, such as EAE, UAE, UHP-assisted extraction, MAE and superfine grinding extraction, in order to improve the yield of LPs and reduce the extraction time. These extraction methods are all processed based on the HWE method, which can be useful from different angles according to different extraction needs. In addition, to further improve the biological activity of LPs and broaden their application scope, researchers have also modified LPs according to their structures. For example, LPs could be both extracted and structurally modified by enzymatic techniques. In this process, the extraction efficiency of LPs was improved. At the same time, the further modification of the structure also enhanced the immune regulation and anti-tumor activities of LPs. These results had a certain relationship with the type, dosage and concentration of enzymes. LPs are mainly composed of Glc, Ara, Man and Gal, which have different types of glycosidic bonds due to their different structures. Structures such as → 6)-Gal-(→ 1 and → 4)-β-Glc → 1 are often present in homogeneous LPs. Studies on biological activities have demonstrated that LPs have a variety of biological activities, including immunomodulatory, anti-oxidant, anti-tumor, prebiotic and anti-inflammatory, which provides a solid foundation for its application in the positive intervention of physiological dysfunction.

However, in general, there are still some shortcomings in the current research on LPs. For example, (1) LPs obtained by modern methods of extraction and purification are still crude LPs, which mostly belong to a class of macromolecules with diverse structures. We lack more clearer and more targeted extraction and purification methods. As a result, many studies have had many difficulties in characterizing their structures, and many higher-level structures are difficult to be elucidated. (2) the research on structural characteristics and activity is not in-depth. We need to carry out high-level structural analysis and structure-biological activity evaluation of LPs, as well as extensive research on the cellular and molecular mechanisms of biological activity. The research results of structure-activity relationship can guide the direction of molecular modification of LPs and provide theoretical support for the design, research and development of LPs agents. Furthermore, more *in vivo* experiments are also needed, which will help to better understand the functional effects of LPs and provide new ideas for further development of natural resources. (3) Although LPs, as natural plant polysaccharides, have no toxic side effects and high affinity to the human body, in order to better apply them to the protection and treatment of human diseases, we still lack systematic and credible quality control standards for LPs. Therefore, the quality evaluation research of LPs and their related products is indispensable to ensure the efficacy and safety of the product. The physical and chemical properties of this polysaccharide are also essential for quality control through quantitative and qualitative analysis.

In summary, the bioactive polysaccharides derived from longan fruit pulp are well-known and widely used in Asia as part of traditional diets and adjuvant therapeutic agents. In the past decades, people have been focused on the composition analysis, structural characterization and biological activity of LPs. LPs can be potentially employed in alternative medicine fields due to its unique physicochemical properties, structural diversity and biological effects, and has a broad application prospect as an immune stimulant or adjuvant anti-cancer agent. Therefore, this paper mainly reviews the LPs from the above aspects, which is helpful to establish a better application of the functional effects of LPs and provide new ideas for the further development of natural resources, for further scientific research and commercial development of LPs.

## Author contributions

XY: methodology, investigation, and writing—original draft. ZC: validation and writing—review and editing. JZ: formal analysis and software. CH: resources. SZ: data curation. XL: visualization. YQ: supervision and funding acquisition. CZ: conceptualization and project administration. All authors contributed to the article and approved the submitted version.

## Funding

This work was supported by the National Natural Science Foundation of China (Grant No. 81603309), National Natural Science Foundation of China (No. 81603281), Health Commission of Sichuan Province (21PJ108), National Interdisciplinary Innovation Team of Traditional Chinese Medicine (Grant No. ZYYCXTD-D-202209), China Postdoctoral Science Foundation (No. 2021M690488), and the Xinglin Scholar Research Promotion Project of Chengdu University of Traditional Chinese Medicine (Grant no. QNXZ2018020).

## Conflict of interest

The authors declare that the research was conducted in the absence of any commercial or financial relationships that could be construed as a potential conflict of interest.

The reviewer YN declared a shared affiliation, with no collaboration, with one of the authors ZC, to the handling editor at the time of the review.

## Publisher's note

All claims expressed in this article are solely those of the authors and do not necessarily represent those of their affiliated organizations, or those of the publisher, the editors and the reviewers. Any product that may be evaluated in this article, or claim that may be made by its manufacturer, is not guaranteed or endorsed by the publisher.
